# Analysis of policy implications and challenges of the Cuban health assistance program related to human resources for health in the Pacific

**DOI:** 10.1186/1478-4491-10-10

**Published:** 2012-05-06

**Authors:** Augustine D Asante, Joel Negin, John Hall, John Dewdney, Anthony B Zwi

**Affiliations:** 1 Human Resources for Health (HRH) Knowledge Hub, School of Public Health and Community Medicine, The University of New South Wales, Sydney, NSW, 2052, Australia; 2 Sydney School of Public Health, The University of Sydney, NSW, 2006, Sydney, Australia; 3 Centre for Clinical Epidemiology and Biostatistics, School of Medicine and Public Health, Faculty of Health, The University of Newcastle, Callaghan, NSW, 2308, Australia; 4 School of Public Health and Community Medicine, The University of New South Wales, Sydney, NSW, 2052, Australia; 5 School of Social Sciences, Faculty of Arts and Social Sciences, The University of New South Wales, Sydney, NSW, 2052, Australia

## Abstract

**Background:**

Cuba has extended its medical cooperation to Pacific Island Countries (PICs) by supplying doctors to boost service delivery and offering scholarships for Pacific Islanders to study medicine in Cuba. Given the small populations of PICs, the Cuban engagement could prove particularly significant for health systems development in the region. This paper reviews the magnitude and form of Cuban medical cooperation in the Pacific and analyses its implications for health policy, human resource capacity and overall development assistance for health in the region.

**Methods:**

We reviewed both published and grey literature on health workforce in the Pacific including health workforce plans and human resource policy documents. Further information was gathered through discussions with key stakeholders involved in health workforce development in the region.

**Results:**

Cuba formalised its relationship with PICs in September 2008 following the first Cuba-Pacific Islands ministerial meeting. Some 33 Cuban health personnel work in Pacific Island Countries and 177 Pacific island students are studying medicine in Cuba in 2010 with the most extensive engagement in Kiribati, the Solomon Islands, Tuvalu and Vanuatu. The cost of the Cuban medical cooperation to PICs comes in the form of countries providing benefits and paying allowances to in-country Cuban health workers and return airfares for their students in Cuba. This has been seen by some PICs as a cheaper alternative to training doctors in other countries.

**Conclusions:**

The Cuban engagement with PICs, while smaller than engagement with other countries, presents several opportunities and challenges for health system strengthening in the region. In particular, it allows PICs to increase their health workforce numbers at relatively low cost and extends delivery of health services to remote areas. A key challenge is that with the potential increase in the number of medical doctors, once the local students return from Cuba, some PICs may face substantial rises in salary expenditure which could significantly strain already stretched government budgets. Finally, the Cuban engagement in the Pacific has implications for the wider geo-political and health sector support environment as the relatively few major bilateral donors, notably Australia (through AusAID) and New Zealand (through NZAID), and multilaterals such as the World Bank will need to accommodate an additional player with whom existing links are limited.

## Background

Small island nations such as those in the Pacific region face considerable challenges in delivering comprehensive packages of primary health care and specialist medical services to their populations. While the effects of climate change and particularly rising sea levels remain the immediate concern of many island states, the poor health outcomes in several of these nations is gaining increasing attention from donor agencies and national governments [1]. Life expectancy at birth in many Pacific Island Countries (PICs) is in the early to mid 60s and lags substantially behind the Western Pacific average of 74 years [2]. Difficulties in accessing basic health care are exacerbated in many countries by the dispersed nature of the population and the rugged terrain. Mortality levels for mothers and newborns in particular remain high in several countries [3]. Pacific Island Countries are undergoing demographic and epidemiological transition with ageing populations and rising incidence of non-communicable diseases especially diabetes in several countries [4]. Table 1 presents a summary of selected health-related indicators for seven Pacific Island Countries and Australia (for comparison).

**Table 1:** Health-related indicators, selected Pacific Island countries and Australia

**Indicators**	**Fiji**	**Kiribati**	**Nauru**	**Papua New Guinea**	**Solomon Islands**	**Tuvalu**	**Vanuatu**	**Australia**
Population (millions 2008)*	0.8	0.1	0.01	6.6	0.5	0.01	0.2	21.0
GNI per capita (ppp US$ 2008)*	4270	3660	3433**	2000	2580	3213**	3940	34040
Life expectancy at birth (2008)*	70	67	60	62	70	64	69	82
Infant mortality/1000 live births (2008)*	16	38	36	53	29	30	27	4
Under-5 mortality/1000 live births (2008)*	18	48	45	69	36	36	33	5
Maternal mortality/100 000 live births (2005)^	210	56^^	-	470	220	-	150^^	4
Diabetes mellitus prevalence estimate (%, 2010)*^	9.1	6.6	30.9	2.1	2.3	13.9	2.4	7.2
Total health expenditure per capita (ppp US$ 2007)^^^	169	358	812	65	123	150	145	3357
Government health expenditure per capita (ppp US$ 2007)*	118	301	575	53	113	149	111	2266

Sources: *WHO World Health Statistics 2010 [5]. ^WHO World Health Statistics 2009 [3]. *^International Diabetes Federation 2010 [6]. ^^^UNDP Human Development Report 2010 [7]. ** World Statistics Pocketbook 2009 [note: figures in current US$] [8]. ^^Figures from UNICEF (http://www.unicef.org/infobycountry) and represent country reported deaths for 2003-2008 [9].

The need to scale up services in order to improve health outcomes in the Pacific has been identified by countries and their development partners. However, as in many developing countries, there are several obstacles to scaling up health service delivery in the Pacific region, one of the most important of which are inadequate numbers of skilled Human Resources for Health (HRH).

HRH are widely perceived to be central to the performance of health systems and the achievement of the Millennium Development Goals (MDGs) [10-12]. The difficulty of ensuring the availability and quality of HRH in low and middle income countries has been recognised by development agencies and was confirmed by the Kampala declaration and agenda for global action which emerged from the Global Forum on Human Resources for Health held in 2008 [4]. Fifty-seven countries, including a number of PICs, were identified in the 2006 World Health Report as having “critical” health worker shortages, which collectively reflected a global shortage of 2.4 million doctors, nurses and midwives [10]; a number of PICs are among them. On average, PICs have 1 doctor per 1000 population compared to about 2.5 doctors per 1000 population for Australia and New Zealand [5]. These shortages have been driven by insufficient training opportunities, migration and attrition [13,14]. Health ministers of countries in the region and their development partners have acknowledged the need to improve the quantity and quality of human resources for health [15]. Table 2 provides health workforce numbers and density in selected PICs.

**Table 2:** Health workforce numbers and density by selected Pacific Island Countries: 2000-2009

**Country**	**Doctors**		**Nurses and midwives**	
	**Number**	**Density (per 10 000 Population)**	**Number**	**Density (per 10 000 Population)**
Australia^	68 689	33	272 741	130
Fiji	380	5	1660	20
Kiribati	20	2	260	30
Nauru	10	8	63	48
Papua New Guinea	275	1	2841	5
Solomon Islands	60	1	630	13
Tuvalu	10	9	50	45
Vanuatu	30	1	360	17

Source: WHO World Health Statistics 2010 [5]. ^Australia Institute of Health and Welfare 2010 [16,17] (Note: figures are for 2008. The density per 10 000 was obtained by dividing the figures by total population of 21 million).

### The Cuban health assistance program

One of the interventions to address HRH shortages in Pacific island nations has been the provision of Cuban health personnel as a component of bilateral health assistance programs. Cuba maintains the largest health cooperation program in the world with more than 38 000 Cuban health workers in over 74 countries while hosting more than 20 000 students studying medicine in Cuba from 60 countries. Overall, some 185 000 Cuban medical professionals have served in 103 countries around the world [18-21]. The Cuban assistance program involves sending doctors on multi-year contracts to countries with shortages to support health care delivery. In addition Cuba offers scholarships to students from those countries to study medicine in Cuba. Upon graduation, these students are expected to return to their countries and the practising Cuban doctors in the countries are withdrawn. Cuba also maintains broader cooperation with several countries that go beyond the provision of health workers and sending of students to Cuba and includes support for domestic medical education. In countries as diverse as the Gambia, Haiti, Timor Leste and Yemen, Cuba has been involved in the creation of medical faculties supported by Cuban academics [22,23]. Additionally, specialist medical training has been provided by Cuban medical brigades in countries such as Timor-Leste [24]. In some countries the Cuban medical assistance program also includes training local people as nurses and nursing assistants [25].

Cost sharing arrangements associated with the Cuban program differ from country to country with wealthier host countries expected to pay more for services of the Cuban doctors. In some cases the services of Cuban doctors are provided in exchange for other resources: Venezuela, for example, agreed to provide 53 000 barrels of oil per day at a reduced price to Cuba in 2000 after Cuba agreed to treat Venezuelan medical patients at no cost and also to supply around 12 000 Cuban doctors and technicians to work in the country [26]. Other countries such as Argentina and South Africa make contributions to the salaries of Cuban doctors they host [27]. In South Africa, Cuban doctors with 10 or more years experience working in government hospitals and institutions in 2007 were reportedly paid a salary of between US$ 3800 - US$ 4400 per month with about 37% of the salary paid directly to the Cuban government [28,29].

The Cuban health assistance program has been most prominent in Africa and the Americas as part of south-south or regional cooperation. In Africa, the Cuban health assistance program dates back to 1963 when a Cuban medical brigade was sent to Algeria. During the years of the anti-apartheid struggle, Cuba provided support to Namibia and Angola. Today, countries such as Ghana, South Africa and Uganda have all hosted Cuban doctors. South Africa welcomed its first group of 92 Cuban doctors in 1996 and a year later 11 medical academics arrived to provide medical training locally. In 2005 there were 777 students from sub-Saharan Africa studying in medical schools across Cuba [30]. In the Americas, countries such as Brazil, Guatemala and Venezuela have received medical assistance from Cuba [31]. In 2003 for example, 550 Cuban health workers, nearly half of whom were women, worked in 20 health areas in 16 departments of Guatemala [32]. Over 30 countries in the Americas have received Cuban health assistance.

International solidarity is one of the key principles underpinning the Cuban health assistance program. This is evidenced by Cuban medical assistance to countries faced with disasters such as the earthquakes in Chile in the 1960s and 1970s, hurricanes in Nicaragua and Guatemala in the late 1990s and tsunamis in Indonesia and Sri Lanka in December 2004 [32]. An assessment of Timor-Leste’s ability to maintain health services during a period of internal political instability and violence highlighted the important support role of the Cuban Medical Brigade, but also brought to the surface relationship and communication issues that deserved attention [33]. The Cuban health assistance program is also situated in a broader context of south-south cooperation associated with the non-aligned movement born out of the Cold War [24]. As a member of the non-aligned movement, Cuba persistently canvassed for unity and cooperation among developing countries to counter what Fidel Castro saw as an unjust international economic order of the time. Cuban assistance to Africa generally can be understood in this context [30].

### Cuban health assistance program in the Pacific

Cuba began formal engagement with Pacific Island governments to discuss the establishment of bilateral health assistance programs in 2008. The engagement coincides with efforts in the region to achieve the millennium development goals (MDGs) and to promote universal coverage of health services. The arrival of Cuban doctors in the Pacific has aroused considerable interest among health policy makers and analysts in the region. The issue was discussed and debated at the Pacific Ministers of Health meeting in Port Vila, Vanuatu in March 2007 and at the Pacific Human Resources for Health Alliance (PHRHA) meeting in Nadi, Fiji in October 2009. Despite the interest, however, very little has been published on Cuban activities in the region with regards to the approach, scope and scale of the health assistance program and its impact. This paper aims to fill this gap by reviewing the Cuban health assistance program in the Pacific and analysing its implications for health policy generally, and the supply of HRH, in particular. It first outlines the current level of involvement of Cuban health workers in Pacific Island Countries and the training of Islander medical students in Cuba before analysing the policy implications.

## Methods

A combination of desk reviews and stakeholder consultations was employed to gather information. We reviewed both published and grey literature on health workforce in the Pacific including health workforce plans and human resource policy documents. Data about current numbers of Cuban doctors working in PICs were obtained from a mapping of HRH resources in the Pacific undertaken by the HRH Knowledge Hub at the University of New South Wales [14] and other online resources. The mapping exercise was intended to generate baseline data on the current HRH situation in the region. It was undertaken between November 2008 and March 2009 using a semi-structured questionnaire administered mainly through telephone interviews and email communications. We also gathered primary information through consultations with health officials from PICs.

## Findings

### Evolution of the Cuban health assistance program in the Pacific

Diplomatic relationships between Cuba and the countries in the Pacific were largely initiated in the early 2000s, except for Vanuatu, where such relations began in the early 1980s. In 2004 cooperation between Nauru and Cuba led to the arrival of 11 Cuban doctors. However, their contracts were terminated ahead of schedule due to language difficulties. Over the next few years, bilateral agreements between Cuba and a few PICs were signed and some Cuban doctors arrived in the region. However, these relationships were made more concrete in September 2008 when the first Cuba-Pacific islands ministerial meeting was held in Havana and representatives from ten Pacific countries - Fiji, the Federated States of Micronesia, Kiribati, Nauru, Papua New Guinea, Samoa, Solomon Islands, Tonga, Tuvalu, and Vanuatu - attended [34]. Table 3 provides a chronology of the evolution of the Cuban health assistance in the Pacific by country.

**Table 3:** Chronology of the Cuban health assistance program in the Pacific

**Country**	**Key dates and events**
Fiji	· 2003 - Fiji and Cuba initiate diplomatic discussions
	· 2005 - Fiji and Cuba establish diplomatic relations and identify medicine as an area of cooperation
	· August 2010 – Six Fijian students leave for Cuba to study medicine
Kiribati	· September 2002 - Kiribati and Cuba establish diplomatic relations
	· 2006 – 15 Cuban medical personnel arrive in Kiribati
	· 2007 – 23 students from Kiribati leave to study medicine in Cuba
Nauru	· First Pacific island country to benefit from Cuban health assistance program
	· May 2002 – Nauru and Cuba establish diplomatic relations
	· 2004 – 11 Cuban doctors arrive in Nauru but contracts terminated after 18 months
	· October 2007 – Signs bilateral collaboration agreement with Cuba on health, education, trade and other sectors
Solomon Islands	· December 2002 – Solomon Islands establishes diplomatic relations with Cuba
	· March 2007 – Cooperation agreement signed between the governments of Solomon Islands and Cuba
	· February 2008 – First batch of 25 Solomon Islands students departs for medical training in Cuba
	· June 2008 – First two Cuban doctors arrive in Solomon Islands and are joined by seven more the following year
	· July 2008 – Second batch of 25 Solomon Islands students departs for medical training in Cuba
	· December 2009 – Third batch of 25 Solomon Islands students departs for medical training in Cuba
Tonga	· June 2002 – Tonga and Cuba establish diplomatic relations
	· October 2009 – Three Tongan students arrive in Cuba to study medicine
	· December 2009 – Tongan Prime Minister travels to Cuba to strengthen diplomatic ties
Tuvalu	· September 2008 – Prime Minister visits Cuba
	· October 2008 – One Cuban health specialist arrives in Tuvalu
	· February 2009 – Two more Cuban doctors arrive in Tuvalu
	· December 2009 – 20 Tuvalu students studying medicine in Cuba
Vanuatu	· 1983 – Relations with Cuba initiated
	· Sept 2008 – 17 Vanuatu students depart for Cuba to start medical training
	· 2008 – Six Cuban doctors arrive in Vanuatu

### Numbers and distribution of Cuban health personnel in PICs

In September 2010, there were a total of 33 Cuban health personnel working in Pacific Island Countries. These are mainly medical doctors and a few medical technicians. Four main PICs - the Solomon Islands, Vanuatu, Kiribati and Tuvalu - have substantial Cuban health personnel presence and considerable numbers of medical students studying in Cuba (Table 4).

**Table 4:** Cuban health personnel in selected PICs and medical students from PICs studying in Cuba, 2010

**Country**	**Number of Cuban Health Personnel in Country**	**Number of Medical Students from PICs studying in Cuba**
Solomon Islands	10	75
Vanuatu	2	37
Kiribati	16	20
Tuvalu	5	30
Nauru	-	9*
Tonga	-	3
Fiji	-	6
**Total**	**33**	**177**

Source: HRH Hub (UNSW) 2009 [14]; *Nauru National Sustainability Development Strategy 2009 Review [35].

Solomon Islands has moved further than most of its Pacific neighbours in embracing the Cuban health assistance program. After signing a cooperation agreement with Cuba in 2007, the Pacific island nation currently has 10 Cuban health personnel (mainly doctors) in-country and 75 students studying medicine in Cuba. This, in part, reflects the need for doctors in the country; the doctor per population ratio according to WHO was 1 per 10 000 in 2000-2009 [5]. Fiji, on the other hand, has been reluctant to accept the Cuban program; no Cuban health personnel are currently working in Fiji and the country only recently (August 2010) took up six of the offered 20 scholarships for medical education in Cuba. This may reflect the less acute shortage of doctors in Fiji than in other PICs [36].

### Costs of Cuban medical aid to Pacific Island Countries

There is very limited data on the costs associated with the Cuban health assistance program in the Pacific. Anderson [27] reported that generally countries that accept Cuban health aid provide accommodation, food, workplace and a monthly allowance of between US$ 150-200, while the Cuban government maintains the doctors’ regular salaries. In the Solomon Islands it is reported that the government pays an allowance of about US$ 300 per month per Cuban health personnel while the Cuban government pays for salaries [37]. Similarly, in Vanuatu, the government reportedly pays a small allowance to the Cuban health personnel and in addition provides accommodation and return airfares. The Cuban government pays their salaries [34]. Pacific governments also make small contributions towards the maintenance of their students in Cuba. According to the Fijian Permanent Secretary for Public Service, the Fijian government provides return airfares for its students studying in Cuba while the Cuban government provides tuition, accommodation, meals, textbooks, monthly allowance and medical/dental assistance to the students [38]. Similar arrangements whereby host countries pay small allowances to Cuban doctors in-country and provide return airfares to students in Cuba are believed to be in place in other PICs. One article estimated that the cost to a Pacific island health ministry to employ a developed country doctor to fill a shortage was more than double that of hosting a Cuban doctor [39].

### Impacts of Cuban medical aid to Pacific Island Countries

There are no official reports from governments of PICs on the impact of Cuban health personnel on health indicators in the region. However a few media reports have attributed improvements in health outcomes to in-country Cuban health personnel. A report by Radio New Zealand stated that the infant mortality rate in Kiribati had been reduced by about 80% following the arrival of the Cuban health personnel with the rate dropping from 50 per 1000 to 9.9 per 1000 [40]. A Cuban news report in mid 2009 states that the three doctors in Tuvalu had attended 3496 patients, saved 53 lives, assisted 76 labours, and performed 11 caesarean sections and 47 surgeries [41]. While the accuracy of such radio reports has not been confirmed (figures do not reflect indicators in Table 1), they suggest potentially valuable contributions to health care delivery by the Cuban health personnel. Clearly, however, these health systems have simultaneously undergone other changes and attribution of improvements to any particular intervention should be undertaken with caution.

## Discussion

### Size and scope of the Cuban health assistance program in the Pacific

The Cuban initiative in the Pacific is relatively new and still evolving, hence, it is difficult to obtain up-to-date data on numbers and distribution of Cuban doctors on the ground. Complicating this is the existing problem of lack of accurate health workforce data in Pacific Island Countries [14]. Although some countries have reasonably detailed and up-to-date workforce statistics including figures on non-national health workers such as the Cubans, the majority of the countries have very limited information on numbers and distribution of health workers. Our review suggests that there are 33 Cuban health personnel working in six PICs and nearly 180 students from various Pacific Island Countries studying medicine in Cuba in 2010. Considering the small populations of PICs, this is a substantial commitment and potentially influential with respect to HRH development and health systems strengthening in the region. Given the current number of doctors in some PICs (Table 2), the return of the Pacific island medical graduates from Cuba upon completion of their studies will double the number of doctors in several countries. This will change not only the doctor to population ratio but also the structure of the workforce, particularly the doctor to nurse/midwife ratio, if there is no corresponding increase in nurse/midwife numbers.

Compared to previous and ongoing medical cooperation between Cuba and other countries, the initiative in the Pacific is small in terms of absolute numbers. For example, there has been relatively much larger Cuban health personnel presence in some countries of Latin America, including Guatemala and Venezuela [32]. Despite the limited current presence in the Pacific, however, an increasing number of PICs perceive the Cuban medical cooperation as a cheaper and more effective model for HRH development and may wish to embrace the initiative and deepen engagement with Cuba in the years to come. It is therefore important to establish mechanisms to document its evolution and to enable assessment of impact on health outcomes.

### Concerns about Cuban health personnel on the ground in PICs

Cuban health personnel are reportedly working in four PICs: Kiribati, Solomon Islands, Tuvalu and Vanuatu. Of a total medical workforce in those countries of around 120, approximately a quarter (33) are Cuban. Anecdotal evidence regarding their contributions to health service delivery in those countries is for the most part favourable [40]. However, some concerns have been expressed about clinical competence, quality of care and language. In discussions relating to participation in the Cuban medical assistance program, the PNG Doctors Association, for example, raised issues about the quality of practice of Cuban medical practitioners working in PICs and mentioned poor clinical standards as a reason to oppose the recruitment of Cuban doctors to PNG [42]. This, however, contrasts with insights from countries such as Indonesia [43], Solomon Islands [44] and Kiribati [41] where Cuban health personnel have reportedly made important contributions to improving health outcomes.

The opposition of the PNG Doctors Association to the Cuban medical cooperation mirrors that of medical associations in other countries hosting Cuban doctors. In Venezuela, Villanueva and de Albornoz [45] report stiff opposition from the Venezuelan Medical Council (VMC) to Cuban doctors working under the government’s Misión Barrio Adentro programme. In Bolivia, the medical association (Colegio Médico de Bolivia) and the association of unemployed doctors went on strike to protest the presence of Cuban doctors [46]. Such opposition by local medical associations may be motivated by self-interest, inadequate consultations, misunderstanding of the Cuban model of health care and language difficulties among others. Medical associations represent professional elites which often protect their interests, status and control against external ‘newcomers’ [47]. It is reported that the Venezuela Medical Council’s opposition to Cuban doctors was, to a large extent, based on its opposition to the Chavez government’s social programs which have made medicine ‘cheaper’ for the poor majority and in the process damaged the social position and income of the medical elites [48]. PNG, unlike Venezuela, has no Cuban doctors on the ground but the PNG Doctors Association may be concerned about potential competition which may erode their social status if the government decides to join fellow PICs in recruiting Cuban doctors.

Inadequate consultation with medical associations by governments hosting Cuban doctors may also give rise to such concerns. In Timor-Leste, some district health officers felt that the Cuban health personnel had been imposed on them from above, although they generally were not opposed to the Cuban medical cooperation [33]. The Venezuelan Medical Counci described the Cuban doctors in the country as working illegally because they had not been registered by the Council. Misunderstanding of the Cuban model of health care may lead to concerns similar to that expressed by the PNG Doctors Association. The Cuban family medicine paradigm is centred on prevention and promotion, without disregarding by any means the curative continuum. This integrated and holistic approach, in which medical doctors are not only ‘curing’ but are also working on different aspects of prevention, is not always understood correctly by ‘classical’ medical doctors [49]. In Venezuela and Bolivia, Cuban doctors have been described by some doctor groups as more akin to “herbalists” and “not qualified to be called doctors” due to their involvement in different health promotion and prevention activities [50].

Evidence emerging from some of the PICs hosting Cuban doctors suggests that some Pacific Islanders are concerned about insufficient language skills which make doctor-patient interaction and their effective integration and collaboration with local doctors difficult. Informal discussions between one of the authors of this paper [JN] and key individuals from some host countries in the Pacific confirmed this. The early termination of the contract of 11 Cuban doctors who arrived in Nauru in 2004 according to the Minister for Health of Nauru was partly due to language difficulties [42]. Concerns about insufficient English language skills of Cuban doctors have been expressed in other countries hosting Cuban doctors. A report from Timor-Leste noted that communities there have expressed concern about the language skills, cultural knowledge and sensitivity of the Cuban health workers [33], although this issue has since received considerable attention.

### Concerns about integration of Islander medical graduates trained in Cuba

While the importation of trained and experienced Cuban personnel is a valuable and immediate contribution to health service delivery, the return home of substantial numbers of Islander graduates from Cuba is of greater long term significance. Concerns have been expressed about integration of the students studying in Cuba into the health systems of the various PICs upon completion of their studies. Specifically, some have questioned whether the returning students will be registrable according to existing accreditation rules and what will be the cost of such integration.

As mentioned above, in PNG where the Cuban initiative has not yet been accepted, the medical association has raised issues with the quality of clinical competence and standard of practice of the Cuban brigade. While no such specific concerns have been raised by any of the countries with students in Cuba, this to some extent foreshadows the level of resistance with regards to registration that may await the Cuban-trained Pacific Island doctors. The process by which returned Cuban-trained doctors will be accredited and their standards assessed is unclear and is likely to vary by country. Medical graduates from Cuba are treated differently in different Latin America countries. In Guatemala, graduates from the Latin America Medical School (ELAM) in Havana can practice in the national health system after a year of hospital based social service which enables them to develop skills required to deal with specific health issues of Guatemala [51]. However, in Honduras ELAM graduates could not practice within the national health system following a dispute over accreditation between the Honduras Council of Higher Education and the country’s medical association. The Council of Higher Education reportedly decided to recognise ELAM degrees and validate the one year clinical clerkship done in Cuba but the Honduras Medical Association opposed the decision arguing that all foreign medical graduates must do a year clerkship and another year social service in-country before being allowed to practice [51].

With regards to the cost of integrating the new medical graduates, governments of countries to which graduates are returning may need to allocate substantial additional recurrent budgets for that purpose. The Solomon Islands, for example, currently have about 75 students in Cuba who will be completing their studies within a few years. This means they will be returning home around the same time and the government may need additional revenue to cover their salaries and other recurrent expenditures associated with their operations. As mentioned elsewhere, it is expected by Cuba and countries with students in Cuba that once the local medical graduates return home, they will replace the in-country Cuban personnel who will be withdrawn. While this arrangement is rational, in terms of cost there will still be the need for additional funding as the in-country Cuban doctors receive an allowance which is not as high as the actual salaries of local medical doctors. In the Solomon Islands, it is reported that the government pays about US$ 300 (SBD$ 2250 Solomon Islands dollars) per month as an allowance for each Cuban doctor. However, a local Solomon Islands doctor is paid a salary of about SBD$ 170 000 (approximately US$ 22 700) per annum [39].

### Implications for training providers

There are two main regional medical training institutions in the Pacific, the Fiji School of Medicine (FSMed) and the University of Papua New Guinea Medical School. Privately run and funded medical schools have recently been established in Fiji and Samoa [14]. While small numbers of PIC students study medicine in Australia and New Zealand, by and large it is the two long established medical schools which have provided the majority of the medical graduates for PICs [52]. Currently, there are not enough places in the two medical schools as staffing and resources are over-stretched [52]. The Cuban medical cooperation may therefore be seen as complementing local training efforts. However, if PIC governments deepen engagement with Cuba and opt to train more medical graduates there, an option which is cheaper for both PIC governments and their students, the initiative can affect enrolment in the regional medical schools with consequent implications for their staffing and funding.

Regarding the training needs of the newly graduated doctors returning from Cuba, regional training institutions may have to expand their activities to offer orientation courses for these graduates. As indicated above, one of the major concerns of local medical associations is the quality of clinical training medical students undergo in Cuba. The training program completed by Islander students in Cuba does not include a post-graduate internship or residency. To standardise practice, quality and clinical competence, ministries of health in PICs may mandate a period of in-country orientation for the returnee medical graduates. Additionally, some in-country arrangements will be needed to provide for the supervision, mentoring and continuing education of the returnees. All of these could pose additional burden on training facilities and put further pressure on staff and resources.

Another matter for consideration at some point is the entry of some of the returned doctors into post-graduate specialist training programs. Specialist training opportunities in the PICs are limited and their expansion will call for resources not readily available. It may be necessary to upgrade the qualifications of would-be trainee specialists and mobilise funding to enable them to participate in specialist training programs outside the PICs.

### Implications for donor assistance for health in the region

The wider geo-political and development context of the Cuban assistance program is also relevant. The Cuban engagement comes amidst a crowded development field in the Pacific. In addition to the traditionally prominent actors such as Australia, New Zealand, the World Bank, Asian Development Bank and Japan, new bilateral partners have been providing increasing amounts of development assistance. China [53]; China (Province of Taiwan); and Cuba have all increased their presence in the region. The South Pacific has become a much more crowded neighbourhood. This has implications for harmonisation and coordination of activities as well as raising the possibility of donors being played off against one another. The Cuban model does not have to be seen as separate from other development assistance, and indeed in other parts of the world, Cuban health assistance programs have been funded by various OECD and new donors: Germany supports programs using Cuban doctors in Honduras and Niger; Japan does the same in Guatemala; and South Africa supports Cuban activities in Mali [22]. In 2010, the Australian Foreign Minister expressed Australia’s readiness to collaborate with Cuba citing Cuba’s renowned medical assistance work [54]. This may highlight an openness on the part of the Australian government to the emergence in the region of this form of development assistance for health.

### Opportunities and challenges of the Cuban health assistance program

The Cuban health assistance program in the Pacific provides avenues for the pursuit of national interests and international activities beyond simply the delivery of health care and the training of medical students. These matters are somewhat outside the scope of this paper. So far as the health services and health personnel are directly concerned, the establishment of the program has opened opportunities for Cuban medical personnel to contribute to, and learn from, activities within PIC health systems to the benefit of themselves, PIC health personnel working with them, the island health systems and the people of the PICs.

The Cuban engagement provides PICs with additional numbers of health personnel that several countries desperately need. However, it is not just the increase in numbers of doctors that the Cuban initiative brings but also the mix of specialist and general practitioners which makes it attractive to PICs [44]. For the Solomon Islands, Cuba has committed to sending a total of 40 medical doctors including surgeons, gynaecologists and other specialists [55]. A willingness on the part of Cuban personnel to work in rural and underserved areas is a further attractive feature of the Cuban health assistance program, built on the premise of health as a right rather than a commodity. In line with their medical philosophy of prioritising rural and community health, a significant number of the Cuban brigade work in remote areas to which local doctors often refuse to go [56]. This provides host countries the opportunity to address the maldistribution of health workers between urban and rural areas. It remains to be seen, however, the extent to which the Cuban-trained Pacific Island doctors will commit to working in rural and remote areas once they complete their studies and return home.

The availability of scholarships for medical training in Cuba represents an extremely generous opportunity for some young PIC people to prepare themselves for a professional career, become literate in another language and gain life experience far wider than that of students who complete their medical school course in one of the PIC regional medical schools.

Our discussion has drawn attention to a number of challenges, some of which are being met as Cuban doctors, their counterparts and support personnel work together in the PICs. Others relating to training and subsequent integration of the Islander graduates from medical schools in Cuba need to be worked through carefully by the respective PIC governments. A review of the long experience of Cuban brigades working in developing countries and of countries successfully integrating newly graduated young doctors into the health system of their home country will provide assistance to the PIC health authorities in foreseeing, forestalling and managing these and other challenges as the partnership Cuba-PIC partnership matures.

## Conclusion

The Cuban health assistance program in the Pacific represents a new and important model of development assistance for health in the region and has the potential to have a significant impact on HRH shortages and ultimately on health outcomes. As the interaction between Cubans and Pacific Islanders increases and matures, deeper investigation and assessments will be needed by all parties to adjust programs and to learn lessons for all the actors in the region. This paper is an initial contribution to establishing a baseline for such assessment and to identify the key issues which deserve ongoing attention. We intend to continue documenting and analysing development of the partnership in the PICs and elsewhere in the region and to provide assistance in the preparation and dissemination of material which will contribute to better understanding of its benefits and costs.

## Competing interests

The authors declare that they have no competing interests.

## Authors’ contribution

AA, JN, JH, JD and AZ collaborated on the design and drafting of the manuscript. JH’s contribution is based on his work while Director of the Human Resources for Health (HRH) Knowledge Hub at the University of New South Wales. All authors approve the final manuscript.

## References

[B1] Australian Agency for International Development Tracking development and governance in the Pacific 2009 AusAID, Canberra

[B2] United Nations Development Programme Human Development Report 2009 - Overcoming barriers: human mobility and development 2009 UNDP, Geneva

[B3] World Health Statistics 2009 2009 WHO, Geneva

[B4] The Kampala declaration and agenda for global action 2008 WHO, Geneva

[B5] World Health Statistics 2010 2010 WHO, Geneva

[B6] International Diabetes Federation Prevalence estimates of diabetes mellitus (DM) 2010 - Western Pacific Region 2010 International Diabetes Federation

[B7] United Nations Development Programme Human Development Report 2010–The Real Wealth of Nations: Pathways to Human Development 2010 UNDP, New York

[B8] United Nations Department of Economic and Social Affairs World Statistics Pocketbook 2009 2010 DESA, New York

[B9] United Nations Children’s Fund Information by country (statistics) 2010 UNICEF; 2010. [http://www.unicef.org/infobycountry/kiribati_statistics.html]

[B10] The World Health Report Working together for health 2006 WHO, Geneva

[B11] Chen L Evans T Anand S Boufford JI Brown H Chowdhury M Cueto M Dare L Dussault G Elzinga G Human resources for health: overcoming the crisis Lancet 2004 364 1984 1990 10.1016/S0140-6736(04)17482-5 15567015

[B12] Chong Y-S Tan E-K Midlevel health-care providers key to MDG 5 Lancet 2011 377 9772 1127 1128 10.1016/S0140-6736(11)60166-9 21458059

[B13] Negin J Australia and New Zealand’s contribution to Pacific Island health worker rrain drain Aust N Z J Public Health 2008 32 507 511 10.1111/j.1753-6405.2008.00300.x 19076739

[B14] Human Resources for Health Knowledge Hub Mapping human resources for health profiles from 15 Pacific island countries: Report to the Pacific Human Resources for Health Alliance (PHRHA) 2009 HRH Hub, The University of New South Wales

[B15] Report of the meeting of ministers of health for the Pacific Island Countries 2005 WHO Western Pacific Region, Apia, Samoa

[B16] Australian Institute of Health and Welfare Medical labour force 2008 2010 AIHW, Canberra

[B17] Australian Institute of Health and Welfare Nursing and midwifery labout force 2008 2010 AIHW, Canberra

[B18] Huish R Spiegel J Integrating health and human security into foreign policy: Cuba’s surprising success The International Journal of Cuban Studies 2008 1 1 42 53

[B19] Huish R Kirk JM Cuban medical internationalism in Africa: The threat of a dangerous example Lat Am 2009 53 3 125 139

[B20] Ministry of Foreign Affairs Cuba Statement by Dr. Rafael Bernal Alemany, First Deputy Minister of Culture of the Republic of Cuba 2009 Cubaminrex-Embacuba Ginebra, Geneva [http://www.cubaminrex.cu]

[B21] Reed G Cuba answers the call for doctors Bull World Health Organ 2010 88 5 325 326 20461209 10.2471/BLT.10.010510 PMC2865666

[B22] Ollier L The experience of using Cuban Doctors to support health service delivery 2008 DFID, London

[B23] Dewdney J Martins J Asante A Zwi A Strengthening Human Resources for Health in Timor-Leste: Progress, Challenges and Ways Foward 2009 The University of New South Wales, Sydney

[B24] Anderson T Cuban health cooperation in Timor-Leste and the South West Pacific 2010 The Reality of Aid, Philippines 77 86

[B25] Huish R Kirk JM Cuban medical internationalism and the development of Lartin America School of Medicine Lat Am Perspect 2007 34 157 77 92

[B26] Alvarez J Fiorito J Venezuelan Oil Unifying Latin-America 2005 Stanford University, Stanford CA

[B27] Anderson T Solidarity aid: the Cuba - Timor Leste health program Int J Cuban Stud 2008 2 53 65

[B28] Nieuwoudt S South Africa welcomes Cuban doctors 2011 [http://ipsnews.net/news.asp?idnews=42156]

[B29] Hammett DP From Havana with love: a case study of south-south development cooperation between Cuba and South Africa in the health care sector 2004 University of Edinburgh, Center for African Studies

[B30] Blunden M South-south development cooperation: Cuba’s health programmes in Africa Int J Cuban Stud 2008 1 1 32 41

[B31] Westhoff WW Rodriguez R Cousins C McDermott RJ Cuban healthcare providers in Venezuela: a case study Public Health 2010 124 9 519 524 10.1016/j.puhe.2010.05.008 20713295

[B32] De Vos P De Ceukelaire W Bonet M Van der Stuyft P Cuba’s international cooperation in health: an overview Int J Health Service 2007 37 4 761 776 10.2190/HS.37.4.k 18072320

[B33] Zwi AB Martins J Grove NJ Wayte K Martins N Kelly P Guterres A Traynor D Gleeson P Tarantola D Whelan A Silove D Timor-Leste Health Sector Resilience and Performance in a Time of Instability 2007 The University of New South Wales, Sydney

[B34] Radio New Zealand International Pacific and Cuba meet to discuss co-operation 2008 RNZ [http://www.rnzi.com/pages/news.php?op=read&id=42045]

[B35] Government of Republic of Nauru National sustainable development strategy review 2009 - Social sector 2009 Government of Republic of Nauru, Nauru

[B36] Fiji Ministry of Health, Women, Social Welfare and Poverty Alleviation Annual Report 2008 2009 MoH, Suva

[B37] Action in Solidarity with Asia and the Pacific 2010 [http://www.asia-pacific-action.org/node/62]

[B38] Fiji Ministry of Information Fijian students study medicine in Cuba MoI 2010 [http://www.fiji.gov.fj/index.php?]

[B39] Wasuka E Cuban doctors to fill shortage 2007 Island Business, Suva

[B40] Radio New Zealand International Cuban doctors reduce Kiribati infant mortality rate by 80 percent 2007 RNZI, [http://www.rnzi.com/pages/news.php?op=read&id=33793] 21 September 2010

[B41] Cuban News Headlines Cuban doctors inaugurated new health servicis in Tuvalu a small Pacific island 2010 Cuban News Headlines [http://www.cubaheadlines.com]

[B42] Radio New Zealand International PNG doctors say Cubans not answer to health care problems 2009 RNZI

[B43] Fawthrop T Impoverished Cuba sends doctors around the globe to help the poor 2006 The Sydney Morning Herald, Sydney

[B44] Mamu M Cuban doctors bring ease to our hospital 2008 Solomon Star, Honiara

[B45] Villanueva T de Albornoz SC Venezuelan doctors resent presence of thousands of Cuban doctors in their country Br Med J 2008 336 7644 57918340061

[B46] Feinsilver J Cuban Medical Diplomacy: When the Left Has Got It Right 2006 [http://www.coha.org/2006/10/cuban-medical-diplomacy-when-the-left-has-got-it-right/Feinsilver]

[B47] Shuval J Elitism and professional control in a saturated market - immigrant physicians in Israel Sociol Health Illn 1995 17 4 550 565 10.1111/1467-9566.ep10932701

[B48] De Vos P Limonta D The Venezuelan Medical Council and the Cuban doctors in Venezuela–Replies to Venezuelan doctors resent presence of thousands of Cuban doctors in their country Br Med J 2008 336 57918340061

[B49] De Vos P No one left abandoned - Cuba’s national health system since the 1959 revolution Int J Health Serv 2005 35 1 189 207 10.2190/M72R-DBKD-2XWV-HJWB 15759563

[B50] Briggs CL Mantini-Briggs C Confronting Health Disparities: Latin American Social Medicine in Venezuela Am J Public Health 2009 99 3 549 555 10.2105/AJPH.2007.129130 19150916 PMC2661459

[B51] Giraldo G Cuba’s piece in the global health workforce puzzle MEDICC Review 2007 9 1 44 47 21487358 10.37757/MR2007V9.N1.8

[B52] Watters DAK Scott DF Doctors in the Pacific Med J Aust 2004 181 11/12 597 601 15588177 10.5694/j.1326-5377.2004.tb06478.x

[B53] Hanson F China: stumbling through the Pacific 2009 Lowy Institute, Sydney

[B54] Australian Government Joint press conference with Mr Bruno Rodriguez, Cuban Foreign Minister 2010 Department of Foreign Affairs, Departmental Media Liaison, Perth

[B55] Solomon Times Online Solomon Islands government welcomes Cuban doctors 2009 [http://www.solomontimes.com/news.aspx?nwID=3779] cited 21 September 2010

[B56] Evans RG Thomas McKeown Meet Fidel Castro Physicians, Population Health and the Cuban Paradox Healthcare Policy 2008 3 4 21 32 19377323 PMC2645168

